# Algorithm of High-Risk Massive Pulmonary Thromboembolism with Extracorporeal Membrane Oxygenation

**DOI:** 10.3390/jcm13226822

**Published:** 2024-11-13

**Authors:** Cagdas Baran, Ahmet Kayan, Canan Soykan Baran

**Affiliations:** 1 Department of Cardiovascular Surgery, Heart Center, Cebeci Hospitals, Ankara University School of Medicine, 06230 Ankara, Turkey; cagdasbaran@gmail.com; 2 Department of Cardiovascular Surgery, Kirikkale High Specialization Hospital, 71300 Kirikkale, Turkey; 3 Department of Cardiovascular Surgery, Ankara 29 Mayıs Hospital, 06105 Ankara, Turkey; canansykn@hotmail.com

**Keywords:** pulmonary embolism, extracorporeal membrane oxygenator, pulmonary embolectomy, extracorporeal life support

## Abstract

**Objective**: Massive pulmonary embolism (PE) remains a life-threatening condition, often leading to acute respiratory and cardiac failure. This study evaluates the role of extracorporeal membrane oxygenation (ECMO) as a supportive treatment for high-risk patients undergoing surgical pulmonary embolectomy or catheter-based thrombectomy. **Methods**: Between January 2018 and December 2023, 27 patients with high-risk massive PE were treated at our center. Surgical embolectomy (*n* = 7) and catheter-based thrombectomy (*n* = 5) were performed, with ECMO support (veno-arterial [VA] or veno-arterial-venous [VAV]) initiated preoperatively, intraoperatively, or postoperatively, based on hemodynamic instability. ECMO was used as a bridge to recovery, and outcomes were assessed in terms of mortality, hemodynamic stabilization, and recovery. **Results**: Of the 27 patients, 20 were supported with ECMO, with 7 requiring VA-ECMO intraoperatively due to difficulties in weaning from cardiopulmonary bypass (CPB). Nine patients were later transitioned to VAV-ECMO due to Harlequin syndrome and persistent hemodynamic instability. The in-hospital mortality rate was 18.5% (*n* = 5), with survivors showing significant improvements in hemodynamic and biochemical parameters post-ECMO, including reduced lactate levels, improved right ventricular function, and the stabilization of mean arterial pressure. The mean follow-up time was 10.2 ± 3.9 months, with no late deaths or complications observed. **Conclusions**: ECMO provides effective life support in high-risk patients with massive PE who are undergoing surgical embolectomy or thrombectomy. It stabilizes hemodynamics, improves cardiac and pulmonary function, and facilitates recovery in critically ill patients. Further research is needed to refine patient selection, optimize ECMO timing, and assess long-term outcomes to determine its definitive role in the management of high-risk PE.

## 1. Introduction

Although advances have been made in the diagnosis, therapy, and development of new oral anticoagulants, acute pulmonary embolism (aPE) and its complications remain common, with high mortality and morbidity rates. The global burden of acute pulmonary embolism has remained significant, with high incidence rates worldwide despite these advancements. The hallmark of high-risk aPE is the presence of hemodynamic instability, caused by a significant obstruction of the right ventricular outflow tract due to clots in the proximal pulmonary arteries. This obstruction leads to increased right ventricular afterload, which can result in severe cardiogenic shock, often referred to as the “right heart failure” phenotype of pulmonary embolism. In this patient group, massive pulmonary embolism plays a significant role in fatal outcomes, with mortality rates as high as 65% in some series, resulting from hemodynamic instability, right ventricular failure, and hypoxemia [1,2]. The rapid progression to shock and cardiac arrest underscores the need for timely, aggressive treatment strategies in this patient population. If intravenous trombolysis is contraindicated or failed, emboli-evacuating interventions such as catheter-based trombolysis or surgical pulmonary embolectomy are the only effective therapies. While thrombolysis remains a first-line approach, its use is limited by contraindications (such as recent surgery or active bleeding) and failure in certain patients, especially those with massive or bilateral embolism.

A gap still exists in the decision-making process and implementation of surgical pulmonary embolectomy, which is considered a last resort due to its invasiveness and associated risks. Additionally, the optimal timing for the use of peripheral extracorporeal membrane oxygenation (ECMO) in high-risk massive pulmonary embolism (aPE) patients is still not well defined, even though other treatment modalities are clearly outlined in the 2014 ESC guidelines for the diagnosis and management of acute pulmonary embolism [3]. In recent series, surgical pulmonary embolectomy has been performed not only in patients with a large central clot burden and hemodynamic compromise but also in hemodynamically stable patients with right ventricular dysfunction. This shift has been facilitated by advances in multidisciplinary approaches, improved experience with ECMO implantation, and a better utilization of ECMO support.

In this study, we reviewed our 5-year experience with the effectiveness of surgical pulmonary embolectomy combined with veno-arterial (VA) and veno-arterial-venous (VAV) ECMO configurations. We specifically assessed the timing of ECMO support, evaluating whether it should be initiated preoperatively, intraoperatively, or postoperatively based on the severity of right ventricular dysfunction and oxygenation status in high-risk massive pulmonary embolism patients. This study aims to contribute to the growing literature on ECMO use in this context, by analyzing the outcomes and exploring whether ECMO should be routinely integrated into the management of patients with high-risk massive pulmonary embolism undergoing surgical intervention.

## 2. Materials and Methods

### 2.1. Study Population

From January 2018 and December 2023, 27 patients with massive or submassive PE applied to our service. Patients in the population ranged from 47 to 82 years old (mean, 62.5 ± 2.12) for the survivor group and 78 ± 1.41 for non-survivors, and 11 were men. A total of 27 patients underwent surgical pulmonary embolectomy (*n* = 7) or catheter-based thrombectomy (*n* = 5) with the placement of VA-ECMO (*n* = 20) support as a preoperative requirement, at the Heart Center of the Ankara University School of Medicine. The institutional review board of our hospital approved the study protocol (Date: 31 July 2024, approval number: 2024/470).

**Figure 1:**
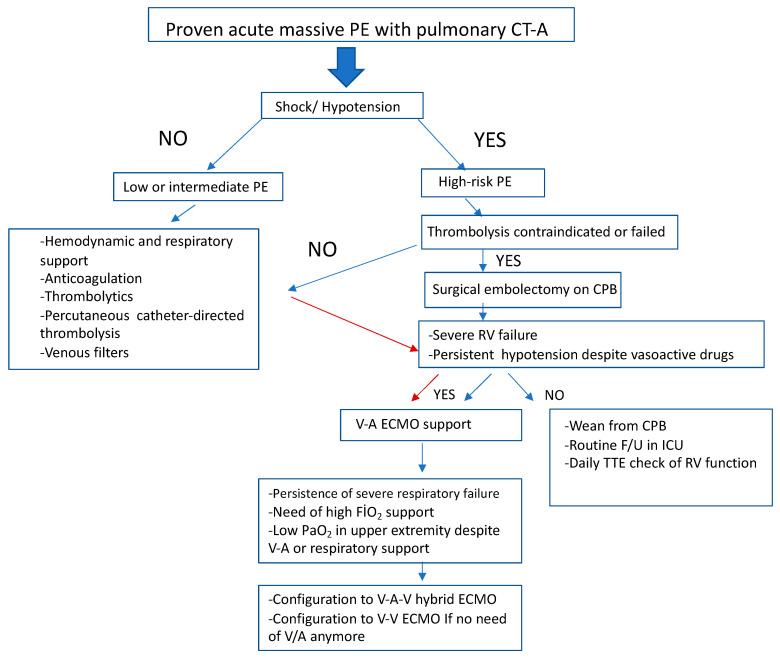
Algorithm for management of patients with massive pulmonary emboli. PE, pulmonary emboli; CT-A, computed tomography angiography; CPB, cardiopulmonary bypass; RV, right ventricle; V-A, veno-arterial; V-A-V, veno-arterial-venous; V-V, veno-venous; ECMO, extracorporeal membrane oxygenation; F/U, follow-up; TTE, transthoracic echocardiography.

Each patient was evaluated by members of the Extracorporeal Life Support (ECLS) team with respect to the reversibility of the pathologic process and appropriateness for ECLS management. All patients had either failed all other medical measures or were too unstable to undergo any further procedures. Overall, inclusion criteria for ECLS for respiratory failure included one or more of the following: PaO_2_/FiO_2_ ratio 100, A-aDO2 gradient 500 mm Hg, transpulmonary shunt 30%, and pulmonary compliance 0.5 cc/cm H_2_O/kg. For cardiac failure, criteria included profound shock despite and after optimal pharmacologic treatment. Extracorporeal life support was instituted either in a veno-arterial (VA) or the common femoral artery and the internal jugular/common femoral veins (VAV). Nine patients required conversion to the VAV-ECMO configuration as a result of Harlequin syndrome on VA-ECMO support. Two patients were placed on life support while in cardiac arrest requiring chest compressions. The management algorithm in patients with massive pulmonary embolism is shown in Figure 1.

Pulmonary embolectomy was performed for patients with high-risk acute pulmonary embolism (aPE) in whom catheter-based or intravenous thrombolytic therapy had failed (*n* = 3) or was contraindicated (*n* = 4) while hemodynamic instability was present. High-risk aPE was defined as aPE if pulmonary computed tomographic angiography (CTA) revealed the presence of bilateral partial occlusion or a unilateral total occlusion of the proximal pulmonary arterial bed and the patient also had hemodynamic instability with hypotension (sBP < 90 mmHg) or cardiac arrest, a diameter ratio of the right ventricle (RV) to left ventricle ≥1 in the echocardiography measurement, or the presence of right heart thrombus. In this study, hypotension was defined as a systolic blood pressure (SBP) <90 mmHg despite adequate fluid resuscitation and pharmacologic support. This definition was used to identify patients with hemodynamic instability secondary to massive pulmonary embolism, who were deemed to be at high risk for adverse outcomes and who subsequently required ECMO support.

### 2.2. Surgical Intervention and Technical Aspects of ECMO Support

All patients in this study group underwent ECMO support, which includes VA-ECMO support for 27 patients primarily. Only 20 patients required preoperative VA-ECMO support due to low cardiac output, although maximum circulatory support including inotropic drugs and mechanical ventilation was provided. We implanted these VA-ECMO machines at the bedside in the intensive care unit via ultrasound-guided percutaneous cannulation and a distal anterograde perfusion catheter.

Seven patients were connected to VA-ECMO intraoperatively due to unsuccessful weaning from CPB with relative distended RV or borderline contractility or global myocardial failure.

The ECMO circuit used in this study consisted of a continuous flow centrifugal pump (JostraRotaflow^®^; Maquet Cardiopulmonary, Rastatt, Germany), oxygenator (JostraQuadrox^®^; Maquet Cardiopulmonary, Rastatt, Germany), and heat exchanger. The system has a CE approval for 14 days of support. The perfusion circuit was primed with Ringer’s lactate, and heparin (1000 IU per liter) was added to the priming dose. The multidisciplinary ECMO team, including cardiovascular surgeons, anesthetists, intensivists, intensive care nurses, and perfusionists, was responsible for the daily management of each patient.

Pulmonary embolectomy was performed under a mild hypothermia (30 °C) beating heart on cardiopulmonary bypass. The pulmonary artery (PA) was incised longitudinally to the bifurcation. A clot was evacuated with tip suction and gallbladder stone forceps, and the main PA branches were gently washed out and aspirated.

Percutaneous Treatment and Failure Criteria:

For patients with high-risk acute pulmonary embolism (aPE) who were unsuitable for or had failed intravenous thrombolytic therapy, we employed catheter-based interventions. The primary percutaneous treatment was catheter-directed thrombolysis (CDT), which involves the administration of thrombolytic agents directly into the pulmonary arteries to dissolve the emboli. In some cases, mechanical thrombectomy was also used as an adjunct, particularly in cases where CDT alone was insufficient or when thrombolysis was contraindicated.

The failure of percutaneous treatment was defined when one or more of the following criteria were met:

Persistent hemodynamic instability despite adequate thrombolysis or thrombectomy. Failure to reduce the thrombus burden as assessed by follow-up imaging (e.g., pulmonary CT angiography or echocardiography). Recurrent symptoms such as severe dyspnea or hypotension despite ongoing treatment. The absence of improvement in pulmonary artery pressures after the completion of the procedure, with sustained elevated right ventricular pressures or persistent right heart strain.

Patients for whom percutaneous treatments failed or were contraindicated proceeded to surgical embolectomy. This approach aligns with the multidisciplinary management strategy for high-risk aPE, as suggested by recent guidelines and studies [4].

Postoperatively, heparin (to partial tromboplastin time, 60 to 70 s, and activating clotting time, 160 to 180 s) was started immediately independent from chest tube drainage because of continuous ECMO support postoperatively in 11 patients. New oral anticoagulant (NOAC) agents were started at 24 to 48 h and continued for at least 6 months. The hematology department evaluated all patients for hypercoagulable states such as antiphospholipid syndrome, lupus, or nephrotic syndrome. All patients were scheduled for pulmonary CTA and a lower limb venous Doppler to determine the discontinuation of NOACs after 6 months.

### 2.3. Management of Patients in ICU and Conversion to VAV-ECMO Support and Weaning Protocols

All patients underwent pulmonary embolectomy with the standard sternotomy approach. Patients were followed up with in the ICU with ventilation support, and the use of VA-ECMO support depended on each patient’s requirement. We always intended to decrease mechanical ventilation support (initial TV 4–6 mL/kg/min, a PEEP of 6–8 cm H_2_O, a FiO_2_ of 0.4 with a PIP ≤ 30 cm H_2_O under a pressure-control mode). In patients who had poor right ventricular function, we used milrinone and dobutamine infusions for 48 h. In intraoperative TEE, if sPAP was greater than 60 mmHg, nitric oxide inhalation was also initiated and continued in the ICU. All patients were examined daily for clinical and echocardiographic changes. PO_2_, PCO_2_, SO_2_, Ph, and lactate levels were followed up via right radial and femoral arterial blood samples. The goals of postoperative care were achieving an acceptable arterial oxygenation via lung protective ventilation, facilitating a further resolution of residual PE, and maintaining a negative fluid balance by diuretics or renal replacement therapy.

ECMO support was initiated as the VA configuration in all patients. We set up the ECMO flow according to the patient’s hemodynamic requirement and respiratory condition and also their daily echocardiographic results which particularly present right ventricular ejection fraction%, pulmonary arterial pressure, the ratio of RV/LV diameters, and the presence of right atrial thrombus. Postoperatively, we tried to wean all patients off respiratory support during ECMO support, so we carried out low-tidal-volume ventilation.

**Figure 2:**
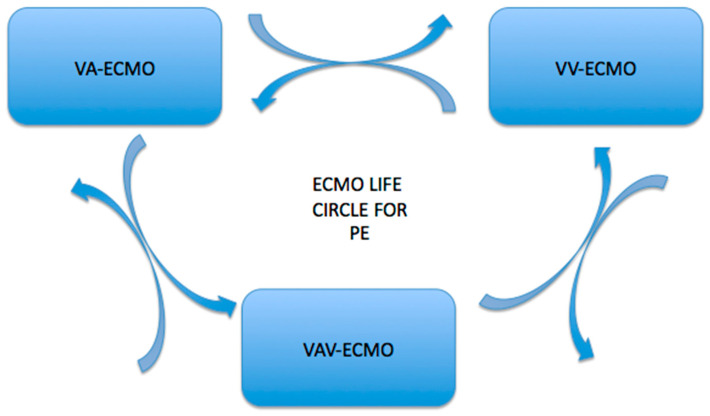
Conversion pathways between veno-arterial (V-A) ECMO, veno-venous (V-V) ECMO, and veno-arterial-venous ECMO (VAV-ECMO). The arrows indicate the potential transitions between these modalities, highlighting the adaptability of ECMO therapy based on patient needs and clinical scenarios.

Switching to the VAV-ECMO configuration for these groups of patients was critical, and this was a multidisciplinary decision made by cardiovascular surgeons, anesthetists, and intensivists (Figure 2). The indication for switching from VA to VAV-ECMO was Harlequin syndrome for 9 patients. An additional 12–17 Fr cannula was percutaneously inserted through the IJV via US guidance and connected to a supplying cannula in a Y-shape fashion. ECMO support was initiated as the VA configuration in all patients who demonstrated severe hemodynamic compromise, defined by profound hypotension (systolic blood pressure <90 mmHg) or cardiac arrest despite maximal medical management. The decision to initiate VA-ECMO was based on the presence of signs of inadequate tissue perfusion, such as elevated lactate levels (>4 mmol/L), severe right ventricular failure (right ventricular ejection fraction <30%), and signs of hypoxemia with a PaO_2_/FiO_2_ ratio <100.

The transition from VA-ECMO to VAV-ECMO was made when patients showed evidence of severe pulmonary dysfunction, specifically in the presence of Harlequin syndrome (differential oxygenation between the two sides of the heart) or ongoing hemodynamic instability due to inadequate oxygenation from VA-ECMO alone. The VAV-ECMO configuration was selected in cases where pulmonary support was deemed necessary in addition to cardiac support, typically indicated by progressive hypoxemia and inability to achieve satisfactory oxygenation despite VA-ECMO support. The decision to convert from VA to VAV-ECMO was made by a multidisciplinary team, including cardiovascular surgeons, anesthetists, and intensivists, based on serial clinical evaluations, echocardiographic findings, and arterial blood gas results.

ECMO support was initiated as the VA configuration in all patients with high-risk massive pulmonary embolism (aPE) who showed evidence of profound hemodynamic instability or respiratory failure that was refractory to standard medical treatment. The decision to initiate ECMO was based on specific criteria related to both hemodynamic and respiratory parameters:

Hemodynamic Criteria:

Severe hypotension (systolic blood pressure <90 mmHg) or cardiac arrest that is unresponsive to pharmacologic interventions (e.g., vasopressors, inotropes).

Right ventricular failure with evidence of low cardiac output despite the use of inotropes, defined as a right ventricular ejection fraction (RVEF) of less than 30% on echocardiography.

Persistent shock despite optimal medical management, including the use of inotropes and vasopressors.

Respiratory Criteria:

Severe hypoxemia (PaO_2_/FiO_2_ ratio <100 or severe acidemia with pH < 7.2) despite maximal ventilation strategies, including high PEEP, prone positioning, or inhaled vasodilators.

Progressive hypercapnia (PCO_2_ > 50 mmHg), indicating inadequate ventilation and failure to correct respiratory acidosis.

ECMO was initiated preoperatively in patients showing signs of deteriorating hemodynamic or respiratory function, particularly in those who had failed medical interventions, such as thrombolytic therapy or catheter thrombectomy. The initiation of ECMO was ideally within the first 6 h after hemodynamic collapse or respiratory failure to prevent irreversible organ damage. In cases where ECMO was required intraoperatively, this decision was based on inability to wean from cardiopulmonary bypass (CPB) due to inadequate right ventricular function or myocardial failure.

Serial echocardiographic assessments, organ perfusion, and arterial pulsatility guided our weaning protocol. We recommenced moderate dose inotropic infusions and re-arranged mechanical ventilatory settings. For the patients with VA-ECMO support, we gradually reduced the flow for weaning according to the left ventricular function, but we mostly did not lower it to less than 1 Lt/min to avoid a possible DPC occlusion which may lead to limb ischemia. We closely monitored the mean blood pressure, central venous pressure, differential arterial blood gases, urine output, and lactate levels. On echocardiography, we aimed to show that the left and right ventricular ejection fraction was above 30% with none to minimal tricuspid regurgitation and aortic valve opening for every beat. We avoided any sign of right or left ventricular distension. We also followed the same strategy for patients with VAV-ECMO support as well.

### 2.4. Statistical Analysis

Demographics and patient characteristics were analyzed using SPSS software (Version 15.0, Chicago, IL, USA). Categorical variables were expressed as percentages and analyzed using the Pearson chi-squared test, while the Fisher exact test was used for nonconsecutive variables. The ANOVA test was used for intragroup comparisons of parameters showing normal distribution in the comparison of quantitative data. The Mann–Whitney U test was employed for consecutive variables, with a significance level set at *p* < 0.05 for a confidence interval of 95%.

Power Calculation:

The required sample size and power calculations were conducted prior to this study using G*Power 3.1.9.4. Based on the expected effect size (Cohen’s d = 0.5), an alpha level of 0.05, and the desired power of 0.80, a minimum sample size of 27 patients was estimated to detect statistically significant differences between groups. This calculation was based on the primary endpoint of hemodynamic stabilization post-ECMO support. The sample size was deemed sufficient to observe clinically meaningful differences in the outcomes of interest. Power analysis indicated that with 27 patients, this study had 80% power to detect significant changes in the mean arterial pressure, central venous pressure, and right ventricular function pre- and post-ECMO intervention.

## 3. Results

A total of 27 patients were analyzed, comprising 22 survivors and 5 non-survivors. The demographic and clinical characteristics of both groups are summarized in Table 1. The average age of survivors was 62.5 ± 2.12 years old, significantly younger than the non-survivors, who had an average age of 78 ± 1.41 years old. The male-to-female ratio was similar in both groups, with six (54.5%) survivors and two (50%) non-survivors being male. Survivors had a higher average weight of 85.5 ± 7.77 kg compared to non-survivors, 77.5 ± 9.19 kg, and a greater height (170.5 ± 13.4 cm vs. 164 ± 12.7 cm). Pre-ECMO right ventricular ejection fraction (RVEF) was 32 ± 5.65% in survivors and 28 ± 5.65% in non-survivors, while left ventricular ejection fraction (LVEF) was also lower in the non-survivor group (49 ± 8.48% vs. 42.5 ± 10.6%). Systolic pulmonary artery pressure (PAP) was similar between the two groups (60 ± 14.1 mmHg in survivors vs. 61.5 ± 19 mmHg in non-survivors). Regarding risk factors, nine survivors (81.8%), for a notable percentage, had a history of smoking, as did four non-survivors (100%). Among the risk factors, deep vein thrombosis (DVT) was present in five (45.4%) survivors and one (25%) non-survivor.

Survivors spent an average of 68.5 ± 16.6 h on mechanical ventilation, significantly less than the 198 ± 31.1 h for non-survivors (*p* = 0.034). The duration of stay in the intensive care unit was similar for both groups, with survivors averaging 4 ± 0.0 days and non-survivors 3.5 ± 0.7 days (*p* = 0.47). The average duration of ECMO support was significantly longer in survivors at 36.5 ± 13.4 h compared to non-survivors at 19.5 ± 2.12 h (*p* = 0.048).

When we looked at the PESI scoring in both patient groups, three (60%) out of five non-survivors were in class IV, and two (40%) were in class V. In 22 survivor patients, 14 (63.6%) were in class V. A comparison of these two patient groups according to the PESI score revealed a statistically significant result (*p* = 0.002). The inclusion of the PESI (Pulmonary Embolism Severity Index) score in the analysis of both the non-survivor and survivor groups provides valuable insights into the severity of the pulmonary embolism and its potential impact on patient outcomes. The fact that the majority of non-survivors were classified in the most severe categories (classes IV and V) reinforces the utility of the PESI as a prognostic tool in assessing high-risk pulmonary embolism patients. In particular, 60% of non-survivors being in class IV and 40% in class V suggests that patients in these categories face considerably higher mortality risks, highlighting the importance of early and aggressive therapeutic interventions.

Interestingly, the majority of survivors (63.6%) were in class V as well, which might initially seem counterintuitive, as this class represents the highest severity. However, this finding may reflect the complexity of managing high-risk massive pulmonary embolism (PE), where certain patients, despite being in the highest PESI risk group, can still survive with timely interventions like ECMO support and surgical embolectomy. This highlights the potential benefit of multidisciplinary approaches in treating high-risk PE patients, as survival can still be achieved even in the highest-risk categories. The statistical significance (*p* = 0.002) observed between these two groups further suggests that the PESI class could be a valuable stratification tool to guide therapeutic decisions and predict outcomes in this patient population. However, it is important to note that while the PESI provides prognostic insight, it is still not a perfect predictor and should be considered alongside other clinical factors when determining treatment strategies.

The overall in-hospital mortality rate was 18.5% (*n* = 5). Mortality was significantly higher in the older age group, with a mean age of 78 ± 1.41 years in non-survivors compared to 62.5 ± 2.12 years in survivors (*p* = 0.04). Among the non-survivors, four out of five patients (80%) had severe preoperative right ventricular dysfunction, as indicated by a right ventricular ejection fraction (RVEF) of less than 25%. Moreover, cardiac arrest prior to ECMO initiation was observed in two patients (40%), who did not survive despite aggressive ECMO and surgical management.

An additional analysis revealed that the duration of ECMO support was shorter in non-survivors, with a mean of 19.5 ± 2.12 h, compared to survivors who had an average of 36.5 ± 13.4 h of ECMO support (*p* = 0.048). The early initiation of ECMO (within 6 h of hemodynamic collapse) appeared to be associated with better survival outcomes, with all survivors receiving ECMO support within this window.

Furthermore, the presence of Harlequin syndrome in three patients was associated with poor outcomes, as they required conversion to VAV-ECMO and experienced severe oxygenation failure despite ECMO support. This subgroup had an in-hospital mortality rate of 50%.

A comprehensive analysis of risk factors for mortality revealed that prior comorbidities, such as chronic obstructive pulmonary disease (COPD) and hypertension, were more prevalent in non-survivors, affecting 60% of the non-survivor group compared to 35% in the survivor group. Additionally, pre-existing deep vein thrombosis (DVT) was identified as a potential risk factor for mortality, occurring in three out of five non-survivors (60%).

Survival rates were also influenced by the timing and modality of surgical intervention. Non-survivors had a significantly higher rate of failure to wean from cardiopulmonary bypass (CPB), with three out of five non-survivors requiring intraoperative ECMO support due to poor weaning from CPB. In contrast, survivors were able to wean off ECMO support more successfully, which correlated with a reduced length of mechanical ventilation and ICU stay.

In the follow-up period (mean 10.2 ± 3.9 months), there were no late deaths or significant complications observed among survivors. However, non-survivors exhibited a higher incidence of postoperative complications, including renal failure and multisystem organ dysfunction.

The hemodynamic and biochemical parameters of patients before and after ECMO support are presented in Table 2. The mean arterial pressure (MAP) remarkably improved from 55.8 ± 13 mmHg pre-ECMO to 67 ± 5.21 mmHg post-ECMO. Central venous pressure (CVP) showed a notable decrease from 15.2 ± 3.1 mmHg to 6.5 ± 1.7 mmHg after ECMO initiation, indicating improved right heart filling pressures.

The right ventricular diameter was significantly reduced from 46 ± 6.5 mm pre-ECMO to 30.4 ± 7.2 mm post-ECMO, reflecting a reduction in RV overload. The ratio of RV diameter to left ventricular (LV) diameter also improved, decreasing from 1.04 ± 0.002 to 0.58 ± 0.16.

Arterial pH increased significantly from 7.14 ± 0.18 pre-ECMO to 7.43 ± 0.13 post-ECMO, indicating better metabolic status. Lactate levels decreased dramatically from 5.9 ± 2.1 mmol/L to 1.95 ± 0.14 mmol/L, suggesting improved tissue perfusion and oxygenation. The partial pressure of carbon dioxide (PCO_2_) decreased from 49 ± 5.2 mmHg pre-ECMO to 39.4 ± 2.3 mmHg post-ECMO, indicating enhanced respiratory function. Systolic pulmonary artery pressure (PAP) decreased significantly from 61 ± 6.2 mmHg to 41 ± 3.8 mmHg following ECMO support.

Right Ventricular Ejection Fraction: The right ventricular ejection fraction (RV EF) improved from 33 ± 3.5% pre-ECMO to 37.2 ± 0.13% post-ECMO, demonstrating enhanced right ventricular function.

## 4. Discussion

The results of our study demonstrate significant improvements in hemodynamic and biochemical parameters following ECMO support in critically ill patients. Specifically, we observed an increase in the mean arterial pressure (MAP) and a decrease in central venous pressure (CVP), indicating enhanced hemodynamic stability post-ECMO. These findings are consistent with prior research, which has shown that ECMO can effectively restore circulatory function in patients with severe cardiac or respiratory failure [5,6]. The stabilization of MAP is particularly critical as it directly correlates with perfusion pressure and subsequent organ function. Recent meta-analytic evidence has shown that exposure to severe hyperoxemia (i.e., oxygen levels exceeding 100% of the target range) during VA-ECMO can have significant negative effects on neurological outcomes and overall survival. In a study by Tigano et al., the authors emphasize that excessive oxygenation can induce oxidative stress and cerebral injury, both of which negatively influence recovery and survival in ECMO patients. This is especially critical during the initial stages of ECMO support, when organ perfusion and oxygenation must be meticulously balanced. Therefore, while maintaining MAP is crucial for perfusion, oxygenation must be carefully managed, with attention to preventing hyperoxemia while ensuring adequate tissue oxygenation [7].

The significant reduction in right ventricular diameter and the improvement in the RV/LV diameter ratio suggest that ECMO support alleviates right ventricular overload. This is crucial, as right ventricular dysfunction is a common complication in patients with severe pulmonary hypertension or acute respiratory distress syndrome (ARDS) [8]. Furthermore, the improvement in right ventricular ejection fraction (RV EF) from 33 ± 3.5% to 37.2 ± 0.13% highlights the positive impact of ECMO on cardiac function, supporting the findings of other studies that reported similar improvements in RV function post-ECMO [9].

Biochemical markers also reflected significant changes, with lactate levels decreasing dramatically, suggesting improved tissue perfusion and oxygenation. Elevated lactate levels are often indicative of tissue hypoperfusion; thus, the reduction we observed aligns with the previous literature indicating that ECMO can enhance metabolic status in critically ill patients [10].

Some studies have shown remarkable results with ECMO alone or ECMO combined with catheter-based thrombolysis in high-risk aPE patients [11,12]. However, more studies are needed to determine the safety and efficacy of these treatment modalities. Our results show that bedside ECMO can be life-saving in patients who, as Corsi et al. have shown, are not able to tolerate instability or other treatments [13]. Pasrija and her colleagues have shown in their work that the use of VA-ECMO ensures the hemodynamic stabilization and normalization of organ functions, allowing for the controlled and non-emergency realization of surgical pulmonary embolectomy. In particular, decompression with VA-ECMO support can significantly improve right ventricular function. In addition, this approach allows an experienced team to evaluate patients under elective conditions and to perform the operation under appropriate conditions after perfusion stabilization [14].

VA-ECMO appears to be an effective approach for optimizing organ function as a bridge to recovery or intervention, demonstrating excellent outcomes. The use of VA-ECMO may enable clinicians to appropriately triage patients with massive pulmonary embolism (PE) based on the recovery of right ventricular function, residual thrombus burden, operative risk, and neurological status. However, there is a need for a prospective analysis that compares the early, aggressive use of VA-ECMO with conventional therapies to further clarify its role in the treatment of PE. This study does have several important limitations. First, it is a retrospective series. The decision to initiate VA-ECMO was not randomized, nor was the final therapeutic intervention determined after ECMO cannulation. Moreover, while the outcomes and survival rates are better than those reported with conventional therapies, definitive claims of superiority cannot be made.

### Study Limitations

This study has several limitations that should be considered when interpreting the results. First, the sample size is relatively small, especially in the non-survivor group, which limits the statistical power of our analysis. Specifically, only five patients in the study cohort did not survive, and this small number makes it difficult to draw definitive conclusions, particularly regarding the impact of ECMO support on mortality. Moreover, almost half of the non-survivor group (two patients) had advanced cancer, which could have significantly influenced their outcomes. Unfortunately, detailed information on the cancer stage and the specifics of cancer treatment was not available, making it challenging to isolate the contribution of cancer to the mortality rate.

Additionally, while we used a retrospective design to evaluate the effectiveness of ECMO in the treatment of high-risk pulmonary embolism (PE), this limits the ability to establish causal relationships or generalize the findings to broader patient populations. Furthermore, the decision to initiate ECMO was not randomized, which introduces a selection bias. We also did not account for all potential confounding factors such as comorbidities, other medications, and the patients’ baseline functional status, which could have impacted both the decision for ECMO and the outcomes.

Due to these limitations, we urge caution when interpreting the results, particularly in the non-survivor group, and recommend further prospective studies with larger cohorts and more comprehensive data collection to confirm our findings.

## 5. Conclusions

In conclusion, our study provides further evidence supporting the use of ECMO as an effective intervention for stabilizing hemodynamics and improving both cardiac and pulmonary function in critically ill patients with high-risk massive pulmonary embolism (PE). ECMO, especially in combination with surgical pulmonary embolectomy or catheter-based thrombectomy, plays a crucial role in the management of patients who fail conventional therapies, such as thrombolysis, or who are hemodynamically unstable.

We observed significant improvements in key hemodynamic parameters, including mean arterial pressure (MAP), central venous pressure (CVP), and right ventricular function, which were consistent with previous studies highlighting the potential of ECMO to support circulatory function in severe PE cases. Additionally, biochemical markers such as lactate levels and arterial pH showed notable improvements, suggesting enhanced tissue perfusion and oxygenation.

However, it is important to note that while the short-term outcomes in this cohort are promising, the small sample size, particularly within the non-survivor group, and the presence of advanced comorbidities such as cancer in some non-survivors limit the ability to draw definitive conclusions about the long-term efficacy of ECMO in this context. As such, future studies should aim to provide more robust data through larger multicenter trials with longer follow-up periods to assess the sustained benefits and potential risks associated with ECMO support in massive PE.

In addition to evaluating long-term outcomes, future research should focus on the development of predictive models to identify patients who are most likely to benefit from ECMO support. These models could take into account not only clinical and hemodynamic parameters but also individual patient characteristics, such as comorbid conditions and response to initial treatments. Such models would help guide clinical decision-making and improve patient selection for ECMO, ensuring that this life-saving intervention is used the most effectively.

In summary, while ECMO shows promise as a critical support strategy in high-risk PE patients, further investigation is needed to optimize its application, refine patient selection criteria, and better understand the long-term impact on survival and quality of life.

## Figures and Tables

**Table 1:** Demographics and characteristics of patients that survived until hospital discharge and those that did not.

	Survivor (*n* = 22)	Non-Survivor (*n* = 5)	*p* Value
Age (years)	62.5 ± 2.12	78 ± 1.41	0.944
Male	6 (54.5%)	2 (50%)	0.21
Weight (kg)	85.5 ± 7.77	77.5 ± 9.19	0.344
Height (cm)	170.5 ± 13.4	164 ± 12.7	0.34
Body mass index (kg/m^2^)	25.0 ± 3.4	25.2 ± 3.6	0.456
Pre-ECMO RVEF (%)	32 ± 5.65	28 ± 5.65	0.078
Pre-ECMO LVEF (%)	49 ± 8.48	42.5 ± 10.6	0.09
Systolic PAP (mmHg)	60 ± 14.1	61.5 ± 19	0.566
Smoking history	9 (81.8%)	4 (100%)	0.069
Risk factors			
Immobility	2 (18.1%)	0	0.42
DVT	5 (45.4%)	1 (25%)	0.566
Cancer	1 (9%)	2 (50%)	0.098
Hypercoagulability	1 (9%)	0	0.607
Hormone replacement therapy	1 (9%)	0	0.344
Prior pulmonary embolism	1 (9%)	1 (25%)	0.899
Preoperative CPR	1 (9%)	2 (50%)	0.754
Preoperative ECMO support	3 (27.7%)	2 (50%)	0.37
Intraoperative ECMO support	5 (45.4%)	2 (50%)	0.213
Bilateral occlusion of PAs	7 (63.6%)	4 (100%)	0.198
PESI score, risk classes			0.002
Class I	1 (9%)	0	
Class II	1 (9%)	0	
Class III	2 (18.1%)	0	
Class IV	4 (36%)	3 (60%)	
Class V	14 (63.6%)	2 (40%)	
Postoperative condition			
Re-open/delay wound closure	2 (18.1%)	1 (25%)	0.349
Nitric oxide inhalation	4 (36%)	3 (75%)	0.277
Persistent coma	0	1 (25%)	0.855
Hours of mechanical ventilation	68.5 ± 16.6	198 ± 31.1	0.034
Days in intensive care unit	4 ± 0.0	3.5 ± 0.7	0.47
Duration of ECMO support (hours)	36.5 ± 13.4	19.5 ± 2.12	0.048
Weaned from ECMO	22 (100%)	0	0.187

Data presented as *n* (%) or mean ± standard deviation. DVT, deep venous thrombosis; CPR, cardiopulmonary resuscitation; ECMO, extracorporeal membrane oxygenation; PA, pulmonary artery; PAP, pulmonary artery pressure; RVEF, right ventricle ejection fraction; LVEF, left ventricle ejection fraction.

**Table 2:** Patient characteristics before and during ECMO support.

	Pre-ECMO (*n* = 27)	VA-ECMO (*n* = 18) + VAV-ECMO (*n* = 9)
MAP (mmHg)	55.8 ± 13	67 ± 5.21
CVP (mmHg)	15.2 ± 3.1	6.5 ± 1.7
RV diameter (mm)	46 ± 6.5	30.4 ± 7.2
Ph	7.14 ± 0.18	7.43 ± 0.13
Lactate (mmol/Lt)	5.9 ± 2.1	1.95 ± 0.14
ECMO flow	-	3.64 ± 0.25
RV diameter/LV diameter	1.04 ± 0.002	0.58 ± 0.16
RV EF%	33 ± 3.5	37.2 ± 0.13
PC02 (mmHg)	49 ± 5.2	39.4 ± 2.3
Systolic PAP (mmHg)	61 ± 6.2	41 ± 3.8

Data presented as *n* (%) or mean ± standard deviation. VA, veno-arterial configuration; VAV, veno-arterial-venous configuration; MAP, mean arterial pressure; CVP, central venous pressure; RV, right ventricle; ECMO, extracorporeal membrane oxygenation; EF, ejection fraction; PAP, pulmonary artery pressure.

## Data Availability

Data are contained within this article.
